# Diagnostic performance of an immunoassay based on urine exfoliated cell enrichment nanotechnology for upper tract urothelial carcinoma: a retrospective, monocentric study

**DOI:** 10.1186/s12894-022-01122-4

**Published:** 2022-11-25

**Authors:** Xin Wang, Shiwei Zhang, Lang Wu, Baofu Feng, Hongwei Shen, Yuanyuan Gu, Qun Zhang, Feng Fang, Rong Yang, Hongqian Guo

**Affiliations:** 1 Department of Urology, Institute of Urology, Drum Tower Hospital, Medical School of Nanjing University, Nanjing University, 321 Zhongshan Rd, 210008 Nanjing, Jiangsu China; 2 PerMed Biomedicine Institute, 201321 Shanghai, China; 3 Department of Pharmacology, Nanjing Medical University, 210000 Nanjing, Jiangsu China

**Keywords:** Urothelial carcinoma, Upper urinary tract, Urine, Immunocytology, FISH

## Abstract

**Background:**

Noninvasively urine-based diagnostic modalities for upper urinary tract urothelial carcinoma (UTUC) were still lacking. We evaluated the diagnostic value of our previously developed urine-based assay (UTC assay) in UTUC.

**Methods:**

We retrospectively analyzed 90 patients with suspected UTUC and 40 donors without UTUC. Voided urine specimens were analyzed by UTC assay and fluorescence in situ hybridization (FISH). The performance of UTC assay and FISH was compared among the 60 histologically proven UTUC patients and the 40 donors with benign disease.

**Results:**

Of the 60 UTUCs, there were 8 low-grade and 52 high-grade cases. Overall sensitivity for UTC assay and FISH were 85% and 73.3%, respectively (P = 0.116). Specificities for UTC assay and FISH were 92.5% and 95%, respectively (P = ns.). By grade, sensitivities of UTC assay and FISH were 87.5% vs. 37.5% for low-grade (P = 0.119), and 84.6% vs. 78.8% for high- grade UTUC (P = 0.446), respectively. By stage, UTC assay showed significantly higher sensitivity than FISH for detecting non-muscle-invasive UTUC, which were 88.5% vs. 61.5%, respectively (P = 0.025).

**Conclusion:**

UTC assay has good performance for the non-invasive diagnosis of UTUC. UTC assay may improve the diagnosis and surveillance of low-grade or superficial UTUC.

## Background

Urothelial carcinoma (UC) can be divided into lower urinary tract (bladder and urethra) UC and upper urinary tract (renal pelvis and ureter) UC. Upper urinary tract urothelial carcinoma (UTUC) is less common, accounting for 5–10% of UC [1]. However, UTUC is an aggressive urologic cancer with a tendency for disease progression and recurrence. UTUC represent a particular subtype of UC frequently associated with activated FGFR3 signaling, a luminal–papillary phenotype and a T-cell-depleted microenvironment [2]. At diagnosis, UTUC is more frequently invasive than bladder tumor (60% vs. 15–25%, respectively) [3]. Early diagnosis is critical for improving clinical outcomes of UTUC because larger tumor size directly and virtually linearly predicts higher rates of invasive and non–organ-confined renal pelvis urothelial carcinoma [4]. Moreover, conservative endoscopic treatment in suitable patients with low-grade (LG) and early stage UTUC is gaining acceptance, highlighting the need for precise diagnosis and robust surveillance [1]. Thus, accurate diagnosis of UTUC aids much in prognosis and decision-making. However, the diagnosis of UTUC is often challenging due to the specific anatomical structure, which hinders timely treatment.

Currently in clinical practice, diagnostic work-up of UTUC has mainly relied on imaging techniques involving excretory urography, retrograde pyelography, computed tomography urography, urine cytology and ureterorenoscopy combining biopsy. Imaging techniques have a low sensitivity in the detection of flat lesions and are susceptible to interference from other factors such as external compression and blood clot [5]. The sensitivity of conventional cytology is low with respect to tumors of the ureter and renal pelvis. Despite the utility of selective upper tract urinary cytology, false negatives occur even up to 60%, especially unfavorable for LG UTUC [6]. Fluorescence in situ hybridization assay (FISH) which consists of fluorescently labeled probes detecting aneuploidy of chromosomes 3, 7 and 17, as well as loss of the 9p21 locus has demonstrated increased sensitivity compared with conventional cytology, however the sensitivity and specificity varied widely, possibly due to different study designs [5]. Also, FISH-based tests had low sensitivity for detecting LG UTUC [7]. Ureteroscopy with biopsy seems reliable, however, unobvious lesions such as flat tumors and carcinoma in situ are probably underdiagnosed and sometimes obtaining a satisfactory biopsy specimen is difficult. Furthermore, it’s invasive, uncomfortable and at risk for a variety of complications such as bleeding, ureteral perforation, disruption or stricture formation. More importantly, ureteroscopy before radical nephroureterectomy may have adverse effects on oncological outcomes [8].

Previously, we developed and validated a non-invasive urine assay that accurately detected UC in bladder, namely “UTC assay”. The UTC assay using voided urine samples showed 80.4% sensitivity and 97.3% specificity for the detection of UC [9]. Since the bladder UC and UTUC share histologically and cytologically similarities [5], we hypothesized that UTC assay could be a useful tool for UTUC diagnosis. The aim of the present study was to evaluate the efficacy of UTC assay for detecting UTUC in comparison to FISH.

## Methods

### Study patients

**Fig. 1:**
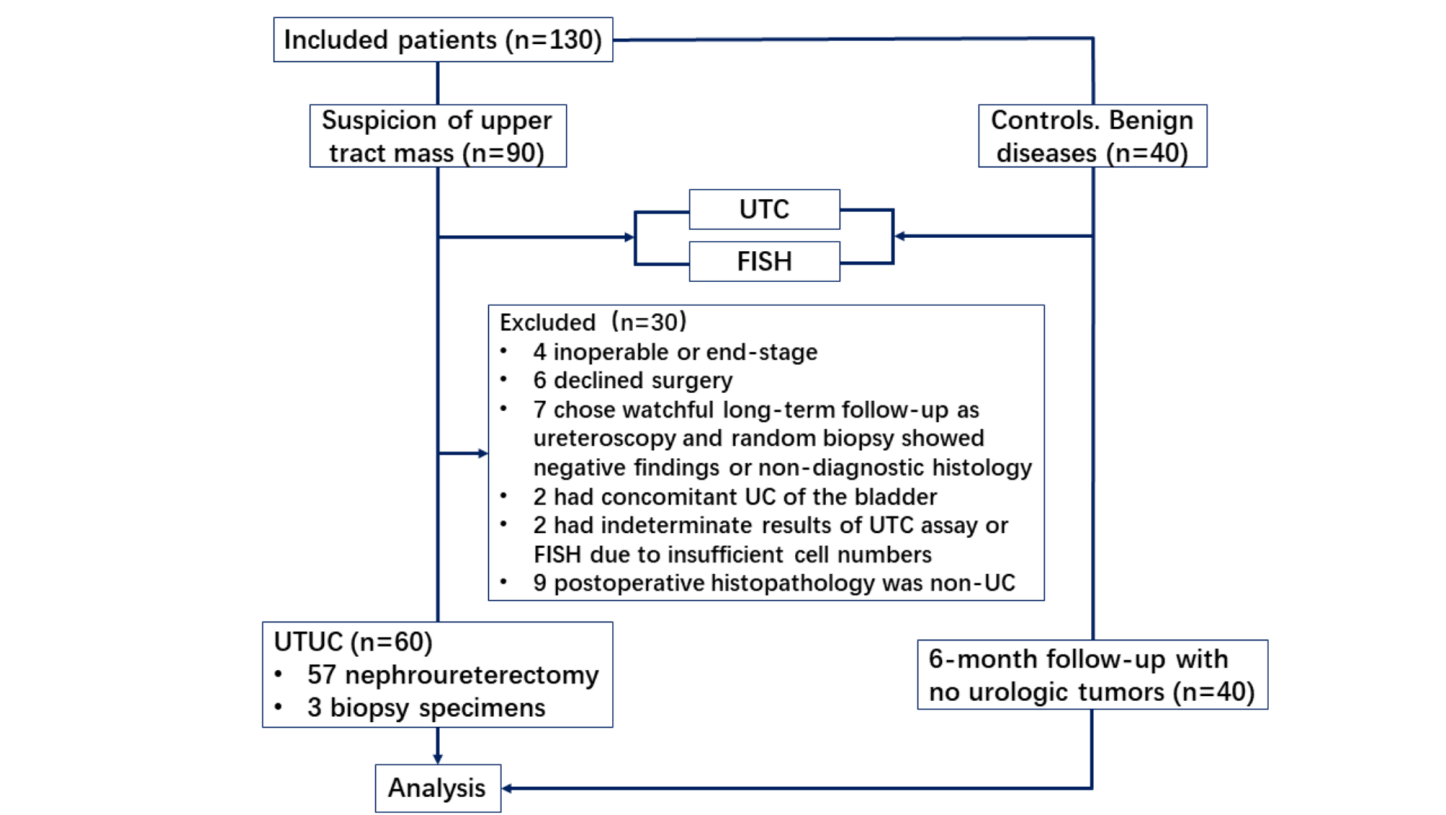
Study flow chart. UC = urothelial carcinoma, UTUC = upper urinary tract urothelial carcinoma

We conducted a retrospective and single center study to compare the performance of UTC assay with that of FISH for UTUC diagnosis. We collected the data of patients suspected of UTUC who underwent both UTC assay and FISH between June 2018 and December 2020. Radiographic (Ultrasound/CTU/MRU) abnormalities such as mass, thickening, filling defects; hematuria from upper tract; or hydronephrosis with atypical obstruction were regarded as abnormal findings suggestive of UTUC. In addition, urine samples from 40 volunteers with no evidence of malignant disease were collected and analyzed by both UTC assay and FISH. These patients were followed up for 6 months by ultrasound (Fig. 1). The written informed consent forms were received from patients prior to inclusion in the study. The study was approved by the ethics committee of Nanjing drum tower hospital and was performed according to the Declaration of Helsinki principles.

### Urine sample collection

The first morning voided urine specimens were collected for UTC assay and FISH. The urine samples for both tests were collected before biopsy, surgery, or any other manipulation. About 100 mL morning urine was collected for UTC assay, and another 200 mL morning urine was collected for FISH. The FISH evaluation was processed according to the routine of the pathology department of our hospital by a senior pathologist and the UTC assay was done in a separate laboratory with specialty training. The reviewers for UTC assay and FISH were not aware of clinical data and also blind to this study. Histologic evaluation of tissue samples obtained from biopsy specimens or surgical resection served as the “gold standard”. The pathologic staging was reported according to the AJCC 8th TNM staging system.

### UTC assay

**Fig. 2:**
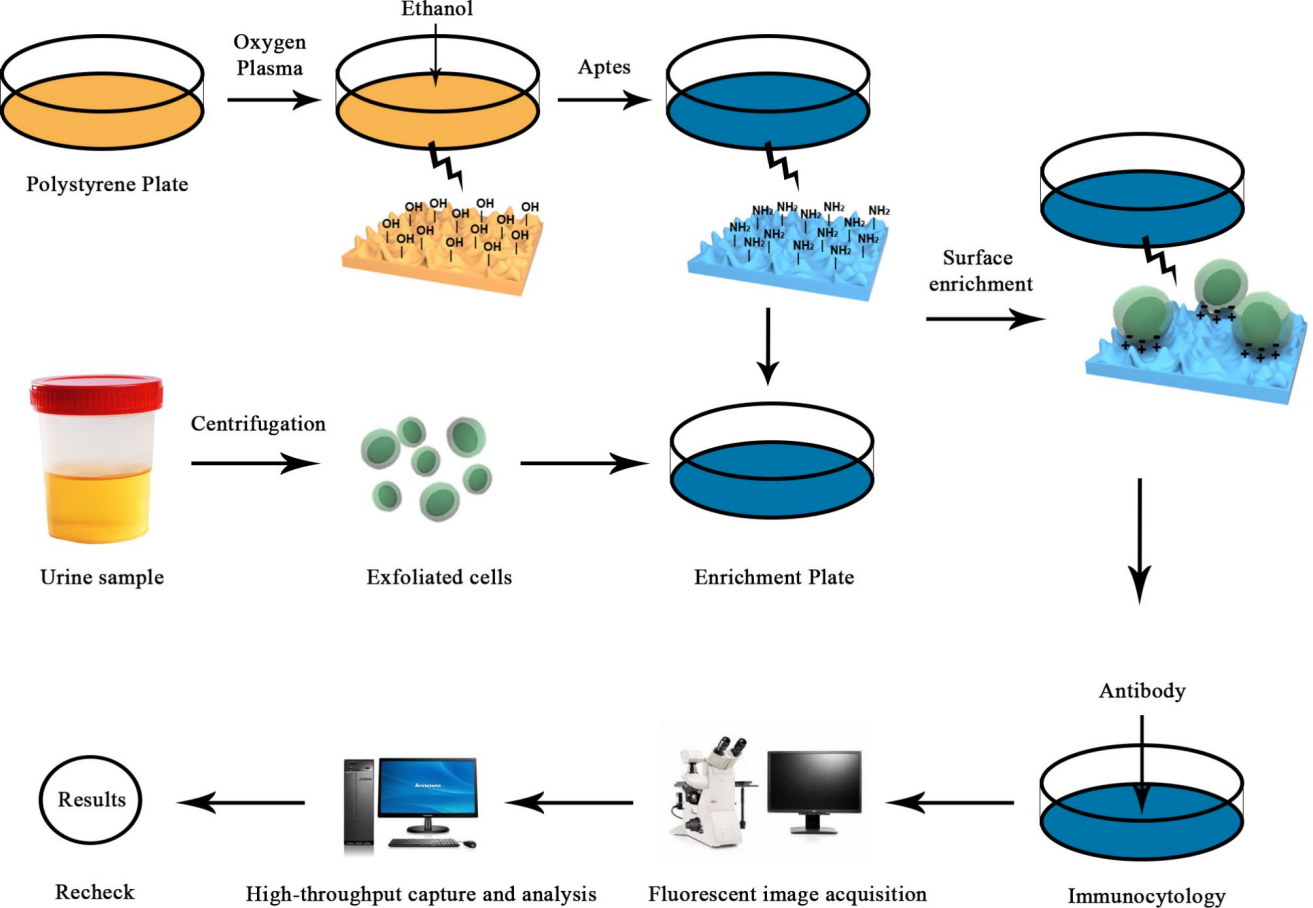
Schematic diagram of UTC assay detection workflow

UTC assay was performed following the reported method as previously described [9] (Fig. 2). Briefly, voided urine samples were stored at 4 °C and centrifuged at 200×g for 10 min within 1 h after collection. Pellets were resuspended by 3 mL FPBS (PBS containing 2% fetal bovine serum) and saved in sample storage tubes transferring to the laboratory. The pretreated samples were then centrifuged at 200 g for 5 min, after which 1 mL FPBS was used to resuspend cells. For the hematuria samples, the depletion of red cells by 1× Lysis Buffer was implemented. The resulting product was added to the prepared nanostructured substrates, then cultured at 37 °C for 1 h followed by 4 °C for 10 min. The supernatant was then removed and the acquired cells were treated with 1 mL 4% paraformaldehyde at 4 °C for 10 min. Next, pre-cooling methanol was used to treat cells for 10 min at -20 °C. Cells were washed by PBS three times and blocked by 5% non-fat milk for 30 min at room temperature afterwards.

Subsequently, the obtained cells were immunostained with CK20 (eBioscience), CD45 (eBioscience), and CD11b (Abcam). Nuclei were stained with 4’,6-diamidino-2-phenylindole (DAPI) (Sigma). CK20 was used as the main marker to detect UC cells that exfoliated in voided urine in this study. CD45 and CD11b were used to mark WBCs and myeloid derivatives, respectively. Cells exhibiting CK20^+^DAPI^+^CD45^−^CD11b^−^ were identified as tumor cells. Finally, Cytell Cell Imaging System (GE Lifesciences) was used to high-throughput capture the stained samples. The supporting software was used to analyze and count cells. The cut-off value of UTC assay is identical to that in our previous report, considering urine tumor cell number ≥ 1 per sample as a positive result [9].

### FISH

200 mL qualified urine sample was centrifuged at 600 g for 15 min, cell pellets were resuspended by collagenase B, after a water bath at 37˚C for 20 min, the sample was centrifuged again at 600 g for 5 min, and then remove the supernatant. Cell pellets were resuspended by 10 mL hypotonic potassium chloride solution and incubated at 37˚C for 20 min. Next, it was fixed in 3:1 (v/v) methanol: glacial acetic acid, then sedimented at 600 g for 5 min, and after the supernatant removed, cell pellets were fixed in another 5 mL 3:1 (v/v) methanol: glacial acetic acid for 10 min and centrifuged at 600 g for 5 min and then pipette the suspended cells again. Drop the cells on the glass slides and treat at 56 °C for 30 min. The glass slides were then incubated at 37˚C with pepsin solution and rinsed by 2× saline-sodium citrate (SSC) solution twice. The slides were then gradient‑dehydrated in ice-cold 70, 85 and 100% ethanol and dried naturally.

Probes comprised centromere enumeration probes (CEPs) of chromosomes 3, 7 and 17, and locus-specific identifier probes to the locus 9p21 of the tumor suppressor gene p16 (LBP Medicine Science & Technology Co., Ltd., Guangzhou, China). 10ul probe mixture was then added to the slide and hybridized overnight at 42˚C after denaturation. Hybridized area was mounted with coverslips and sealing film. The slides were then washed twice in 2× SSC solution at room temperature, gradient‑dehydrated in ice-cold 70, 85 and 100% ethanol, and naturally dried in the dark. After stained with DAPI, slides were covered with coverslips and placed in the dark for 15 min.

Slides were observed under fluorescence microscope (BX51, Olympus) by a cytopathologist. The image was captured by a cold charge coupled device (CCD) camera controlled by the FISH point counting analysis software Imstar 3.0. For each hybridization zone, 100 cells were analyzed. Overlapped cells, damaged cells, and phyllodes nucleus were not counted, neither were weak hybridization signals. Intracellular hybridization signals close to each other were counted as one signal. Fluorescence signal of CSP3 (green), CSP7 (cyan), CSP17 (red), and GSP P16 (red) were observed. The positive judgment standard of the combined probes was: ≥4 cells show 2 or 3 increased color signals in hybridization zone I, or ≥ 4 cells show 1 increased color signal in hybridization zone Iand simultaneously presenting ≥ 12 cells with loss of red signal in hybridization zone II.

### Statistical analysis

Statistical analysis for evaluating the differences between UTC assay and FISH was performed using two-sided Fisher’s exact test in 60 patients with histopathology proven UTUC and 40 cases in the control group. When not applicable, χ2 test was performed instead. P < 0.05 was considered to indicate a statistically significant difference. All analyses were performed using IBM SPSS Statistics 25.0.

## Results

**Fig. 3:**
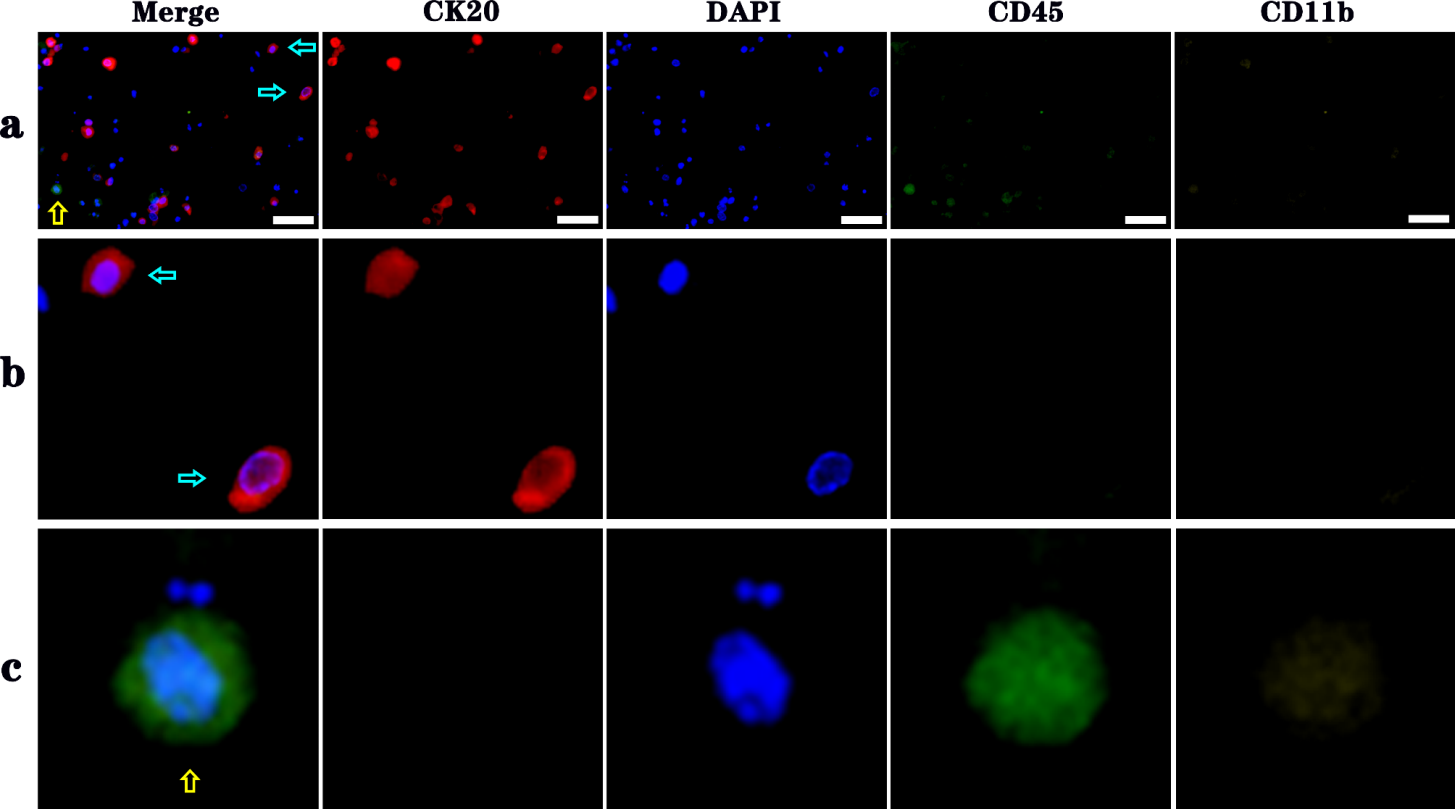
Example of the positive UTC assay result from a voided urine sample of UTUC. **a** UC cells (cyan arrows) and WBC (yellow arrow) were detected by immunofluorescence staining of cells captured on NS. Scale bar: 50 μm. **b** Magnified view of UC cells indicated by cyan arrows in (a), showing CK20^+^DAPI^+^CD45^−^CD11b^−^. **c** Magnified view of the WBC cell indicated by yellow arrow in (a), showing CK20^−^DAPI^+^CD45^+^CD11b^−^.

**Fig. 4:**
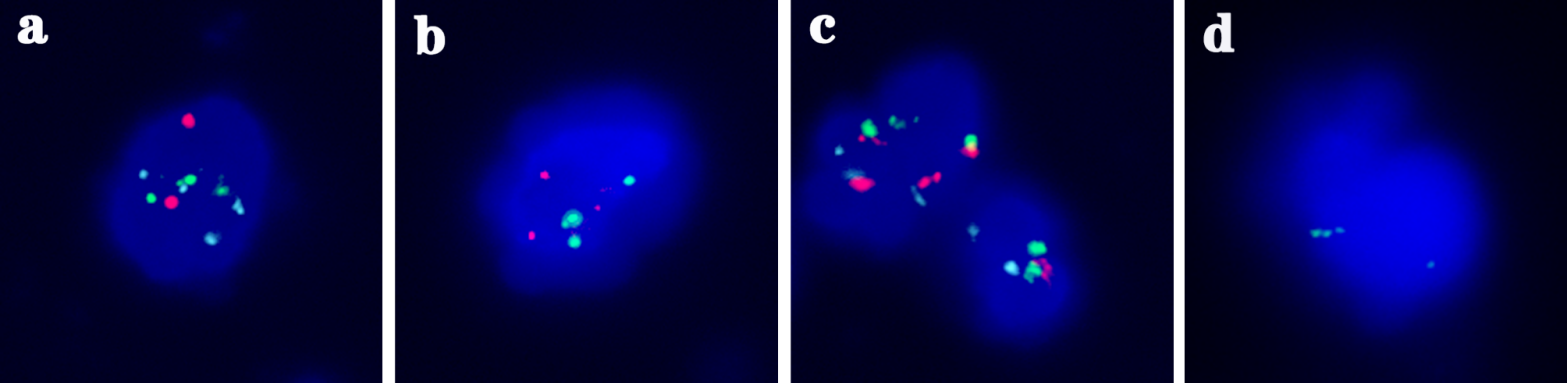
Examples of abnormal FISH images for each probe from voided urine samples of UTUC. **a** Polyploidy of chromosomes 3 (green) and 7 (cyan). **b** Aneuploidy of chromosomes 3 (green) and 17 (red). **c** Polyploidy of chromosomes 3 (green), 7 (cyan) and 17 (red), top left. **d** Homozygous deletions of P16 (9p21) (0 red), and amplification of chromosomes 3 (green)

A total of 90 patients suspicious for UTUC were evaluated by UTC assay and FISH during the same period. However, 21 patients were excluded for the following reasons: 4 patients were inoperable advanced cases receiving conservative treatment, 6 patients declined surgery, 7 patients chose watchful long-term follow-up as ureteroscopy and random biopsy showed negative findings or non-diagnostic histology, 2 patients had concomitant bladder UC, 2 patients had indeterminate results of UTC assay or FISH due to insufficient cell numbers. Moreover, 9 patients found with postoperative histopathology other than UC were also excluded, i.e. 1 mucinous adenocarcinoma, 1 malignant spindle cell tumor, 1 papillary renal cell carcinoma, 1 chromophobe renal cell carcinoma, 2 smooth muscle hyperplasia, 1 submucosal cavernous hemangioma, and 2 ureteral polyp. Finally, the voided urine specimens from 60 patients with pathology-confirmed UTUC (57 nephroureterectomy and 3 biopsy specimens) were considered as experimental group for the sensitivity study. The patient demographics are shown in Table 1. Of the 60 patients, 38 (63.3%) were men, and 22 (36.7%) were women. The median age at the time of diagnosis was 69 years (46–89 years). 32 were located in ureter, 24 were detected in renal pelvis, and 4 invaded both renal pelvis and ureter. Representative examples of positive UTC assay and FISH results are shown in Figs. 3 and 4, respectively.

**Table 1:** Patient Characteristics

Clinicopathologic features	UTUC	Control	
Patients, n	60	40	
Population	Asian	Asian	
Gender, n (%)			
Male	38 (63.3)	27 (67.5)	
Female	22 (36.7)	13 (32.5)	
Median age, years (range)	69 (46–89)	47 (25–71)	
BMI (kg/m²), mean ± SD	25.3 ± 1.7	26.1 ± 2.4	
Current smoking, n (%)	25 (41.7)	12 (30)	
Hypertension, n (%)	21 (35)	9 (22.5)	
Diabetes mellitus, n (%)	11 (18.3)	4 (10)	
Pathology		Diagnosis	No.
Low-grade	8 (13.3)	kidney stones	15
High-grade	52 (86.7)	ureteral stones	15
Stage, n (%)		renal cyst	5
pTa	7 (11.7)	pyelonephritis	3
pTis	2 (3.3)	UPJ obstruction	2
pT1	17 (28.3)	-	
pT2	5 (8.3)	-	
pT3	26 (43.3)	-	
pT4	3 (5)	-	

UTUC = upper urinary tract carcinoma; UPJ = ureteropelvic junction; no. = number; BMI = Body Mass Index

Sensitivity, specificity and predictive values are listed in Table 2. The overall sensitivity of UTC assay to detect UTUC was 85.0% (51/60), whereas that obtained by FISH was 73.3% (44/60) (P = 0.116). UTC and FISH results were concordant in 41 (68.3%) of 60 biopsy-proven UTUC cases, including both UTC and FISH positive in 38 (63.3%) and both UTC and FISH negative in 3 (5%). In other words, 57 (95%) cases have at least one positivity of UTC assay and FISH.

**Table 2:** Sensitivity, specificity, PPV, and NPV of UTC assay and FISH

	UTC assay	FISH	*P* value
	**No. accuracy/Total (%)**	**No. accuracy/Total (%)**	
Stage			
Non-MI (pTa, pTis, pT1)	23/26 (88.5)	16/26 (61.5)	0.025
MI (pT2-T4)	28/34 (82.4)	28/34 (82.4)	ns.
Grade			
Low	7/8 (87.5)	3/8 (37.5)	0.119
High	44/52 (84.6)	41/52 (78.8)	0.446
Overall sensitivity	51/60 (85)	44/60 (73.3)	0.116
Overall specificity	37/40 (92.5)	38/40 (95)	ns.
PPV	51/54 (94.4)	44/46 (95.7)	ns.
NPV	37/46 (80.4)	38/54 (70.4)	0.247

FISH = fluorescence in situ hybridization; MI = muscle invasive; PPV = positive predictive value; NPV = negative predictive value; ns. = non-significant

The comparison between UTC assay and FISH according to stage and grade is shown in Table 2. Of the 60 UTUCs, 7 had stage pTa disease, 2 had pTis disease, 17 had pT1 disease, 5 had pT2 disease, 26 had pT3 disease, 3 had pT4 disease (Table 1). Thus, the subjects were classified as non-muscle-invasive (pTa, pTis, pT1) in 26 (43.3%) and muscle-invasive (pT2-4) in 34 cases (56.7%). UTC and FISH sensitivities on detecting non-muscle-invasive tumors were 88.5% (23/26) and 61.5% (16/26), respectively (P = 0.025), whereas for muscle-invasive tumors, it was 82.4% for both tests. Strikingly, the UTC assay detected all of the 7 pTa cases, however FISH detected only 3 pTa cases (P = 0.07). Of note, 10 of 16 false-negative samples by FISH were from patients with non–muscle-invasive tumors. For grade classification, the tumor was LG in 8 (13.3%) and high-grade (HG) in 52 (86.7%) patients. Sensitivities of UTC assay and FISH by grade were 87.5% (7/8) versus 37.5% (3/8) for LG (P = 0.119), and 84.6% (44/52) versus 78.8% (41/52) for HG UTUC (P = 0.446), respectively (Table 2). Of note, as listed in Table 3, UTC assay demonstrated to be positive for 4 of 5 false-negative LG cases encountered by FISH.

**Table 3:** UTC assay and FISH results of patients with LG UTUC (n = 8)

Case	Sex	Age	Tumor location	Stage	UTC assay			FISH
					Total cell no.	Positive cell no.	Result	
1	Male	62	Renal pelvis	Ta,N0,cM0	10,962	9	+	-
2	Male	62	Renal pelvis	T1,N0,cM0	5,068	2	+	-
3	Female	83	Proximal ureter	Ta,N0,cM0	65,021	5	+	-
4	Male	57	Distal ureter	T1,N0,cM0	64,503	>100	+	-
5	Male	65	Renal pelvis and ureter	T1,N0,cM0	228,660	5	+	+
6	Male	54	Proximal ureter	T1,N0,cM0	30,031	111	+	+
7	Male	53	Renal pelvis	T1,N0,cM0	4,854	0	-	-
8	Female	82	Distal ureter	T1,N0,cM0	144,584	3	+	+

LG = low-grade; ‘+’ = positive for UTUC; ‘−’ = negative for UTUC; FISH = fluorescence in situ hybridization;no. = number

The 40 patients in control group were all followed up for 6 months by ultrasound and found no evidence of urologic tumors. Diagnosis of the 40 patients in control group consists of 15 kidney stones, 15 ureteral stones, 5 renal cyst, 3 pyelonephritis and 2 UPJ obstruction (Table 1). Median age of them was 47 (25–71) years. The specificity of UTC assay was 92.5% (37/40), and for FISH, it was 95.0% (38/40) (P = ns.). The positive predictive value (PPV) of UTC assay and FISH were 94.4% (51/54) and 95.7% (44/46), respectively (P = ns.). The negative predictive value (NPV) of UTC assay and FISH were 80.4% (37/46) and 70.4% (38/54), respectively (P = 0.247) (Table 2).

## Discussion

Early diagnosis for UTUC has important clinical implications for improving patient outcomes [10]. However, accurate diagnosis is still challenging owing to the difficult accessibility of the upper tract, causing clinical decision-making intractable, especially when radiologic imaging or biopsies are inconclusive or not diagnostic. We have previously identified a urine-based non-invasive UTC assay which detects bladder UC with high sensitivity and specificity [9]. Accordingly, this study aimed to assess the value of the UTC assay as a diagnostic tool for UTUC. Our results show that UTC assay identified UTUC with 85.0% sensitivity in void urine samples, maintaining satisfactory specificity. Furthermore, UTC assay showed excellent performance in subgroups of stage and grade. Remarkably, UTC assay increased the sensitivity rates as compared to FISH across non-muscle-invasive and LG UTUC.

Previously reported data agreed that conventional urine cytology has little value in the diagnosis of UTUC [11]. Although the collection of urine specimens by ureteral catheterization can improve the sensitivity to some degree, the sensitivity of selective upper tract urinary cytology is 53.1%, specifically, 45.6% for LG tumors and 69.9% for HG tumors [6], still not good enough, and catheterization may increase false-positive rate. Voided urine collection is convenient and comfortable, particularly for patients under long surveillance. Therefore, we selected voided urine as the research specimen for UTC assay.

FISH was first reported to be feasible and effective for the diagnosis of UTUC using voided urine specimens by Marín‑Aguilera et al. with overall sensitivity of 76.7% and specificity 94.7% [12]. A few years later, Mian et al. reported a FISH analysis with 100% sensitivity using washing samples of the renal pelvis or ureter in a small UC cohort [13]. Three FISH studies reported sensitivities of 78.9%, 84.0% and 85.7% respectively on voided urine specimens [14–16]. Sensitivity of FISH in the present study was somewhat lower (73.3%), the discrepancy might be attributed to the different interpretation standard for positive FISH test (e.g., cut-off value and quality control), difference in patient selection, and more importantly the “voided urine” sample collection method. Indeed, there were even more disappointing sensitivities of voided FISH in previous literature, and some were as low as 52% and 54%, questioning its usefulness for UTUC diagnosis and surveillance [17–18]. It is of note that FISH is much less sensitive in detecting LG UTUC. Our results are consistent with the previous findings that FISH has a low sensitivity in non-invasive and LG tumors [12, 17–18].

One possible reason for the poor FISH results in LG and non-muscle-invasive UTUC is these relatively low-burden tumors are less prone to shedding tumor cells into the collecting system [11]. Correspondingly, our final analysis found that one FISH negative LG case did show genetic abnormalities (p16-, + 17, +7, + 3), but only in one cell, not reaching positive threshold. Likewise, for two of the three both negative (UTC assay and FISH) cases, surgical records indicated twisted and narrowed ureters resulting in procedures failure during preoperative ureteroscopy. Urinary obstruction caused by tumor or inflammation might prevent the dissemination of exfoliated tumor cells to urine [19]. We also speculated that reduced urine flushing due to reduced glomerular filtration rate of the ipsilateral kidney or decreased ureteral motility may further retard cell shedding. Optimization of cell collection either by washing urine or from voided urine is crucial [20]. It was reported that the result of immunocytology was dependent on the technique of slide preparation [21]. One of the advantages of UTC assay is the urine exfoliated cell enrichment nanotechnology. Among the UTC assay treated 60 UTUC voided urine specimens, the median enriched and high-throughput analyzed cell number was 8,384, the most one being 525,468 cells. However, during FISH judgment process, only about 100 tested cells in one analysis sample are observed in the view of microscope in routine process of our pathology department. Thus, UTC assay improves sample collecting from another aspect, that is, greatly increase the number of tested cells. By making full use of the exfoliated cells from upper tract, UTC assay can greatly increase the probability of capturing cancer cells. Meanwhile, after the efficient, strong, and rapid attachment of tumor cells in urine, the nanostructured substrates could retain morphology of the binding cells for further judgement. Another possible explanation is that LG tumors in these patients were generally diploid or near-diploid tumors with relatively few chromosomal abnormalities [22]. Molecular genetic studies suggest that LG pTa tumors have relatively few chromosomal alterations other than deletions of part or all of chromosome 9 [23].

The Immunocyt test which targets the monoclonal antibodies M344, LDQl0 and 19A211 was reported to be sensitive for all grades and stages bladder UC and high sensitivity to LG UTUC [24, 25]. Messing et al. confirmed the performance of Immunocyt for detecting LG, superficial, small tumors [26]. Intrinsically, the UTC assay is an immunocytology method applying immunoreaction against tumor associated antigen CK20 based on cell enrichment nanotechnology and high-throughput analysis. CK20 expression in exfoliated urine cells has been extensively studied either with immunocytochemistry or RT-PCR [21, 27–31]. Bhatia et al. reported that CK20 is an important biomarker that can be used to identify UC in urine cytology smears, especially useful in those cases where the atypical cells are fewer in number or obscured by the inflammatory cells [27]. Buchumensky et al. reported a CK20 RT-PCR-based study of voided urine samples and observed a sensitivity of up to 91% [30]. Importantly, CK20 showed high diagnostic efficiency for LG tumors [28, 32], which may be another important interpretation accounting for high sensitivity of UTC assay in LG UTUC.

Apart from standard treatment of nephroureterectomy with a bladder cuff for UTUC, endoscopic treatment of biopsy-proven LG UTUC in selected patients such as those with solitary kidney, renal insufficiency, bilateral upper tract cancer or clinically low-risk cancer is reasonable [1]. The patients underwent endoscopic ablation and surveillance are the primary candidates to potentially benefit from a urine marker that could predict therapeutic effect and recurrence. The diagnostic dilemmas created by LG urothelial neoplasms of the upper tract are difficult to resolve by conventional cytology, nor FISH [5, 17–18, 33]. J. E. Freund el al evaluated FISH in 1 mL of passively collected selective urine as a triage test for kidney sparing surgery of UTUC, reporting high sensitivity of 90% and a specificity of 80%, however the sample size of the study is small [33]. In contrast, FISH showed disappointing sensitivity for LG UTUC in our study, indicating that the usefulness of voided FISH in this patient population is unsuitable, which is similar to the study of James et al. [18]. The encouraging results of UTC assay in the present study suggest that it is an alternative diagnostic option in selected patients, especially in the surveillance of patients treated endoscopically for LG UTUC.

UTC assay has several additional clinical advantages. The accuracy of the cytology and immunocytology is dependent on cytopathologists’ experience and skills. For FISH, the counting and interpretation process is troublesome. In this regard, UTC assay can reduce interobserver difference by using fully automated high-resolution cell imaging system, which allow maximum analysis of fluorescent staining signal and high-throughput analysis of cell structure. The examination time of UTC assay is shorter as the immunostaining technique is simpler and easier compared with FISH. Last but not least, the cost of UTC assay is lower, which is nearly half of that of FISH.

The limitation of the current study was the retrospective design and relatively small number of patients from a single institution. Our preliminary results warrant further larger and multi-center studies, particularly, for LG UTUC. Additional well-designed prospective studies on UTC assay are needed for validation.

## Conclusion

Our preliminary results suggest that UTC assay provides high sensitivity and specificity and exhibits superior sensitivity for LG and superficial UTUC compared to those of FISH. UTC assay showed its potential to be a noninvasive test for the detection of UTUC. Further larger prospective and multicenter research is required.

## Data Availability

The datasets supporting our findings are presented in the article. The datasets of the current study are available from the corresponding author on reasonable request.

## Abbreviations

UTUC: upper urinary tract urothelial carcinoma
FISH: fluorescence in situ hybridization
UC: urothelial carcinoma
LG: low-grade
HG: high-grade
CTU: computed tomography urography
MRU: Magnetic resonance urography
AJCC: American Joint Committee on Cancer
TNM: tumor-node-metastasis
FPBS: phosphate-buffered saline containing 2% fetal bovine serum
SSC: saline-sodium citrate
CEP: centromere enumeration probe
CCD: charge coupled device
UPJ: ureteropelvic junction
PPV: positive predictive value
NPV: negative predictive value
